# Impact of DNA and RNA Methylation on Radiobiology and Cancer Progression

**DOI:** 10.3390/ijms19020555

**Published:** 2018-02-12

**Authors:** Hsiang-Cheng Chi, Chung-Ying Tsai, Ming-Ming Tsai, Kwang-Huei Lin

**Affiliations:** 1 Radiation Biology Research Center, Institute for Radiological Research, Chang Gung University/Chang Gung Memorial Hospital, Linkou, Taoyuan 333, Taiwan; hgchi@mail.cgu.edu.tw; 2 Kidney Research Center and Department of Nephrology, Chang Gung Immunology Consortium, Chang Gung Memorial Hospital, Chang Gung University College of Medicine, Taoyuan 333, Taiwan; monster_0616@yahoo.com.tw; 3 Department of Nursing, Chang-Gung University of Science and Technology, Taoyuan 333, Taiwan; mmtsai@gw.cgust.edu.tw; 4 Department of General Surgery, Chang Gung Memorial Hospital, Chiayi 613, Taiwan; 5 Liver Research Center, Chang Gung Memorial Hospital, Linkou, Taoyuan 333, Taiwan; 6 Department of Biochemistry, College of Medicine, Chang-Gung University, Taoyuan 333, Taiwan; 7 Research Center for Chinese Herbal Medicine, College of Human Ecology, Chang Gung University of Science and Technology, Taoyuan 333, Taiwan

**Keywords:** radiotherapy, DNA methylation, RNA methylation, epigenetic regulation

## Abstract

Radiotherapy is a well-established regimen for nearly half the cancer patients worldwide. However, not all cancer patients respond to irradiation treatment, and radioresistance is highly associated with poor prognosis and risk of recurrence. Elucidation of the biological characteristics of radioresistance and development of effective prognostic markers to guide clinical decision making clearly remain an urgent medical requirement. In tumorigenic and radioresistant cancer cell populations, phenotypic switch is observed during the course of irradiation treatment, which is associated with both stable genetic and epigenetic changes. While the importance of epigenetic changes is widely accepted, the irradiation-triggered specific epigenetic alterations at the molecular level are incompletely defined. The present review provides a summary of current studies on the molecular functions of DNA and RNA m^6^A methylation, the key epigenetic mechanisms involved in regulating the expression of genetic information, in resistance to irradiation and cancer progression. We additionally discuss the effects of DNA methylation and RNA *N*^6^-methyladenosine (m^6^A) of specific genes in cancer progression, recurrence, and radioresistance. As epigenetic alterations could be reversed by drug treatment or inhibition of specific genes, they are also considered potential targets for anticancer therapy and/or radiotherapy sensitizers. The mechanisms of irradiation-induced alterations in DNA and RNA m^6^A methylation, and ways in which this understanding can be applied clinically, including utilization of methylation patterns as prognostic markers for cancer radiotherapy and their manipulation for anticancer therapy or use as radiotherapy sensitizers, have been further discussed.

## 1. Introduction

Radiotherapy has been established as one of the major treatment options for patients with cancer in the clinic for over 100 years, based on the theory that cancerous regions can be destroyed with targeted ionizing radiation exposure, while normal tissue parts surrounding tumor lesions can withstand and recover after radiotherapy [1]. However, accumulating evidence indicates that a decisive small population of radioresistant cancer cells exhibits stem cell characteristics responsible for tumor initiation, maintenance, and progression. These cancer-initiating cells, designated cancer stem cells (CSCs), are characterized by their potent tumorigenic properties and ability to self-renew [2,3,4]. More importantly, cancer stem cells contribute to radioresistance as well as chemoresistance, and are believed to mediate relapse and recurrence of the disease after therapy [5]. Cancer stem cell markers are reported to be epigenetically regulated [6]. For instance, DNA methylation is linked to several crucial pathways involved in CSC activation, including Wnt (Wingless-type MMTV integration site family)/β-catenin (catenin beta 1), Hh (Hedgehog), and Notch signaling [7]. 

Efforts to improve radiotherapeutic strategies, such as optimization of treatment plans and precision of dose delivery, are not beneficial in some cases, and inter-individual differences in therapeutic activity are commonly observed [8]. Cellular response to radiotherapy is dependent on the molecular composition of cancer cells. However, the availability of predictive biomarkers that can be used to monitor alterations in molecular composition and predict outcomes of radiotherapy is limited [8,9]. Combination of the clinical characteristics of patients with molecular or imaging markers may aid in the identification of prognostic factors in cancer patients treated with radiotherapy [10]. 

Alterations in the epigenetic patterns in the structure and function of chromosomes are heritable events that occur without changes in the DNA sequence. In mammalian cells, epigenetic changes, including DNA and RNA m^6^A methylation, have been implicated in several critical biological roles, including cellular proliferation, differentiation, and development of multiple organisms [11,12]. Dysregulation of epigenetic mechanisms leads to global changes in genomic packaging and specific gene promoter changes that influence the transcription of downstream genes involved in cancer progression [13]. Thus, regulation of epigenetic mechanisms has been established as an emerging strategy for cancer therapy. 

Cell proliferation and survival require tight regulation and propagation of genetic material, which are attacked by both intracellular and extracellular environmental sources of DNA damage. Irradiation is considered a potent DNA damage inducer and epigenotoxic agent [14]. Within the epigenetic parameters, DNA methylation is significantly implicated in the context of radiation biology [14,15]. DNA methylation is one of the known epigenetic mechanisms involved in the regulation of genetic material. Recent studies have additionally demonstrated crucial roles of RNA m^6^A methylation in both irradiation-triggered DNA damage response and radioresistance [16,17]. In the current review, we have provided an overview of the reported roles of DNA and RNA m^6^A methylation in radiobiology and cancer progression. Application of epigenetic regulators and biomarkers in radiotherapy is further discussed. Epigenetic regulators are speculated to contribute to radioresistance and metastasis of tumors. Elucidation of the molecular cues underlying the effects of epigenetic changes following irradiation should facilitate the design and development of effective strategies to improve the therapeutic effects of radiotherapy and prevent cancer recurrence. 

## 2. Roles of DNA Methylation in Radiotherapy and Cancer Progression

### 2.1. DNA Methylation 

DNA methylation is considered the most common mechanism regulating epigenetic events and is closely associated with the progression of several cancer types, including breast, colon, lung, and prostate cancer [18,19]. The process is catalyzed by DNMT (DNA methyltransferases), and usually occurs at the 5′ position of the cytosine ring within the CpG (cytosine guanine dinucleotide) island. To date, five members of the DNMT family have been identified, among which DNMT1 (DNA methyltransferase 1), DNMT3A (DNA methyltransferase 3 alpha), and DNMT3B (DNA methyltransferase 3 beta) have functional activity in mammalian cells. DNMT1 shows up to a 50-fold preference for hemimethylated DNA substrates at the CpG island after DNA replication, and is thus designated “maintenance DNMT”. DNMT3A and DNMT3B generate new methylation patterns on both DNA strands during embryogenesis and development of germ cells. DNA methylation is implicated in gene inhibition during development, and leads to potent X-chromosome inactivation and genetic imprinting. In addition to regulation of gene expression, DNA methylation protects cells from chromosomal instability through inhibition of endogenous retroviral and parasitic repetitive sequences [20]. Additionally, alterations in DNA methylation patterns that appear to contribute to cancer progression have been extensively documented. For instance, DNA hypermethylation of cancer cells may present an alternative complementary mechanism to trigger mutation or silencing of specific genes, consequently leading to acquisition of tumorigenic behavior, including cellular survival and metastasis [13,18]. In addition to DNA hypermethylation, the genetic material of cancer cells undergoes a global decrease in 5-methylcytosine levels. The overall hypomethylation of the genetic material influences intergenic and intronic DNA, repetitive, and transposable elements especially, leading to loss of imprinting, chromosomal instability, and reactivation of endogenous parasitic sequences [21]. Radiotherapy plays a crucial role in the curative treatment of various malignancies, and radioresistance is highly associated with poor prognosis and relapse of cancers [22]. Mounting evidence indicates that irradiation treatment may lead to aberrantly altering DNA methylation patterns, and radiation-induced epigenetic changes may contribute to the initiation of radioresistance [23]. 

### 2.2. Ionizing Radiation and Global DNA Methylation

The effects of radiotherapy on genetic alterations have been extensively documented [24,25,26]. However, epigenetic alterations induced by irradiation, even those causing changes in transcriptional activity and cellular resistance to radiation therapy, remain to be clarified [14,15]. The earliest research interest on the impact of irradiation on DNA methylation was traced back to 1972 when an overall increase in DNA methylation was observed in *Escherichia coli* 15T following irradiation [27]. Subsequent experiments showed that irradiation triggers dynamic changes in DNA methylation, with patterns of DNA hyper- and hypo-methylation observed in the thymus and bone marrow of Wistar rats [28]. Moreover, the level of 5-methylcytosine was significantly decreased in several organs and cancer cell types, including ovarian, lung fibroblasts, HeLa, and neuroblastoma cells [29]. Follow-up studies have suggested that the levels of change in DNA methylation in response to irradiation are dose- and tissue-dependent. For instance, global DNA methylation is decreased in liver thymus, spleen, bone marrow, and mammary gland, but not muscle and lung [30,31,32,33,34]. Irradiation-induced global hypomethylation, in vitro and in vivo, possibly occurs as a result of decreased expression of DNA methyltransferases or methyl-CpG binding proteins, including DNMT1, DNMT3A/3B, MBD2 (methyl-CpG binding domain protein 2), and MECP2 (methyl-CpG binding protein 2) [35,36,37]. These effects are more apparent after fractionated irradiation, and are sex- and tissue-dependent [38], and appear persistent, even after repair of irradiation-triggered DNA damage [33,34,36,39,40]. 

### 2.3. Gene-Specific DNA Methylation as a Potential Predictor of Response to Radiotherapy

As global hypomethylation is linked to malignant transformation and carcinogenesis, DNA hypomethylation triggered by radiation therapy may be utilized as a marker of oncogenic transformation [33,34]. In addition to causing global changes, radiation induces alterations in methylation locus-specific regions [41,42]. DNA methylation at promoter regions of specific genes thus shows prognostic potential, and may present effective markers to predict the outcomes of radiotherapy (Table 1).

#### 2.3.1. Glioblastoma Multiforme (GBM)

The methylation level of the promoter of *MGMT* (*O*^6^-methylguanine-DNA methyltransferase), a radiation-induced gene that encodes a DNA repair enzyme responsible for removing alkyl groups from guanine, was identified as a predictive epigenetic biomarker in glioblastoma [43,44,56]. Patients with hypermethylation of the *MGMT* promoter have been shown to display better survival following adjuvant chemotherapy or radiotherapy [45,57,58]. This finding may be attributable to hypermethylation-driven suppression of *MGMT* expression, and consequent blockage of the inhibitory effects on the chemotherapeutic activity of drugs or irradiation in tumor killing. In contrast to data obtained from patients with glioblastoma, the methylation level of the *MGMT* promoter was associated with poorer prognosis or higher chance of relapse after chemo- or radiotherapy in other solid tumors, such as cervical cancer and non-small-cell lung cancer patients with brain metastasis [46,59]. 

#### 2.3.2. Lung Cancer

Significant DNA hypermethylation of *MGMT* and *CDKN2A* (cyclin-dependent kinase 2A) genes in sputum of uranium miners was previously reported [60]. These genes are frequently hypermethylated and inactivated during tumor progression, particularly in lung cancer [47,61]. Further studies revealed higher methylation levels of *CDKN2A* in lung adenocarcinomas from plutonium-exposed workers, compared to non-exposed workers at MAYAK, a Russian nuclear enterprise [48]. Hypermethylation-driven silencing of *CDKN2A* expression has also been observed in a murine model of radiation-induced thymic lymphoma [49]. Aberrant methylation of *CDKN2A* is therefore proposed as a potentially useful marker to predict tumor cell response to chemo- and radiotherapy. In non-small cell lung cancer (NSCLC), global analysis of CpG methylation has been used to determine the factors associated with epigenetic control of radiosensitivity. In a study by Kim et al. [62], a higher proportion of hypermethylation was observed in radioresistant NSCLC cells, and 1091 differentially methylated genes were identified, among which, 747 were hypermethylated and 344 were hypomethylated. Furthermore, hypermethylated genes were implicated in multiple processes, including regulation of inter- and intra-cellular signaling, while most of the hypomethylated genes were implicated in transcriptional control. Among the genes displaying the most significant differences in methylation, *SERPINB5* (serpin family B member 5) and *S100A6* (S100 calcium binding protein A6) hypermethylation, and *CAT* (catalase) and *BNC1* (basonuclin 1) hypomethylation were implicated in radioresistance of NSCLC. Data from this study suggest that response to irradiation is highly dependent on the overall methylation profile of tumors [62]. 

While these studies clearly suggest a role for epigenetic biomarkers in determining response to radiotherapy, the prognostic value of the potential markers identified requires validation in large patient populations to ensure their clinical utility.

#### 2.3.3. Esophageal Cancer

In esophageal cancer, *CDKN2A*, *RPRM* (reprimo), *CDKN1C* (cyclin dependent kinase inhibitor 1C), *TP73* (tumor protein p73), *RUNX3* (runt related transcription factor 3), *CHFR* (checkpoint with forkhead and ring finger domains), *MGMT*, *TIMP3* (TIMP metallopeptidase inhibitor 3), and *HPP1* (hyperpigmentation, progressive, 1) comprise a marker panel showing decreased methylation in radiation-responsive patients. Conversely, increased methylation of these genes is significantly correlated with poor responsiveness to chemoradiation [63]. Runt-related transcription factor 3 (*RUNX3*), a tumor suppressor that mediates transforming growth factor TGF-β (Transforming growth factor beta) dependent apoptosis [50,51], is reported to be hypermethylated and downregulated in radioresistant esophageal cancer cells. Both *RUNX3* expression and methylation levels in pretreatment specimens may be applied to predict radiosensitivity of esophageal squamous cell carcinomas [52]. 

#### 2.3.4. Cervical Cancer

*TP73*, a member of the p53 family of transcription factors involved in cellular responses to stress and development, is hypermethylated in radioresistant cervical cancers, and significantly associated with silencing of p73 expression [53]. Higher p73 expression is positively associated with radiosensitivity of cervical cancer cells, and may play an important role in regulating the radioresponse of tumors.

#### 2.3.5. Head and Neck Squamous Cell Carcinoma (HNSCC)

In patients with advanced head-and-neck squamous cell carcinoma (HNSCC) treated solely with radiotherapy, promoter hypermethylation-driven silencing of *TIMP3*, an inhibitor of matrix metalloproteinases, and *CDH1* (cadherin 1), a calcium-dependent cell–cell adhesion protein, have been identified as markers to predict better therapeutic outcome [54]. Another study identified five frequently methylated tumor suppressor genes, including *IRX1* (iroquois homeobox 1), *EBF3* (early B cell factor 3), *SLC5A8* (solute carrier family 5 member 8), *SEPT9* (septin 9), and *SKOR2* (SKI family transcriptional corepressor 2), in HNSCC following radiotherapy. Alterations in methylation of promoters of this subset of genes were enriched in pathways implicated in radiation responses, including cell cycle regulation, DNA repair, and apoptosis [42]. A recent study using Human Methylation450 BeadChip, in combination with gene expression profile analysis, identified 84 differentially expressed genes that display differential methylation levels between radioresistant and radiosensitive HNSCC cells. Data from this investigation disclosed significantly increased DNA methylation in radiation-resistant cells. Notably, the differentially methylated and expressed genes in radioresistant cells were implicated in the regulation of integrin-linked kinase and glucocorticoid receptor cascades, fatty acid catabolism, and cell proliferation. Further validation studies indicated that cyclin D2 (*CCND2*), a potent cell cycle regulator, is hypermethylated at the promoter region, and downregulated in radioresistant head and neck squamous cells [55]. 

#### 2.3.6. Nasopharyngeal Carcinoma (NPC)

Radiotherapy is the standard therapy of choice for nasopharyngeal carcinoma (NPC), and aberrant DNA methylation is known to be involved in NPC response to radiotherapy. A recent study reported that inactivation of miR-24 through hypermethylation of its precursor promoter is associated with NPC radioresistance. Furthermore, treatment with 5-aza-2′-deoxycytidine compensated for reduced miR-24 expression and sensitized radioresistant NPC cells to therapy [64]. 

*FHIT* (Fragile histidine triad) gene, a triphosphate hydrolase involved in purine metabolism, is hypermethylated and consequently silenced in established radioresistant oral cancer cells [65]. Further in vivo experiments confirmed that inhibition of DNA methylation of *FHIT* leads to significant resensitization of the radioresistant oral tumors. Furthermore, hypermethylation of the *FHIT* promoter was inversely correlated with its expression, and served as an independent predictor of both overall survival and locoregional control in oral cancer patient samples. These data suggest that expression and hypermethylation-driven silencing of *FHIT* are the determining factors for radiosensitivity in oral cancer. 

#### 2.3.7. Laryngeal Cancer

Another study used radioresistant laryngeal cancer cells established via long-term fractionated irradiation to identify the crucial genes with DNA hypermethylation involved in radioresistance of cancer. Increased methylation levels of promoters of *TOP2A* (DNA topoisomerase II alpha), *PLXDC2* (plexin domain containing 2), *ETNK2* (ethanolamine kinase 2), *GFI1* (growth factor independent 1 transcriptional repressor), and *IL12B* (interleukin 12B) were detected in radioresistant laryngeal cancer cells. Elimination of methylation of *TOP2A* by treatment with the DNA methyltransferase inhibitor, 5-aza-2′-deoxycytidine, not only sensitized resistant laryngeal cancer cells to radiotherapy, but re-activated expression of these genes, clearly supporting the theory that changes in DNA methylation levels contribute to radioresistance of laryngeal squamous cell carcinoma [45].

#### 2.3.8. Breast Cancer

The effects of fractioned ionizing radiation at a dose of 2 Gy with cumulative doses of 10 and 20 Gy on DNA methylation in the human breast cancer cell line, MCF7, were examined [41]. Cells were harvested 48–72 h after the final irradiation, as well as the recovery period of up to 24 days. A subset of genes was differentially methylated in response to radiation treatment. Specifically, increased methylation was observed in *ADAMTS9* (ADAM metallopeptidase with thrombospondin type 1 motif 9), *FOXC1* (forkhead box C1), and *TRAPPC9* (trafficking protein particle complex 9), while the methylation level of *AMIGO3* (adhesion molecule with Ig like domain 3) was decreased in response to radiation. Further in vitro experiments showed significant methylation loss in *FOXC1* and *TRAPPC9* after a recovery period in which irradiated cells displayed regrowth, compared to control cells. As both genes are implicated in cell death control, alterations in their DNA methylation patterns may lead to reduced apoptotic signaling, resulting in regrowth of breast cancer cells after radiation [41]. Antwih et al. [35] globally analyzed DNA methylation changes at >450,000 loci in breast cancer MDA-MB-231 cells subjected to X-ray irradiation. Their findings suggest that that differentially methylated genes (for instance, *AGAP1* (ArfGAP with GTPase domain, ankyrin repeat and PH domain 1), *ARHGEF10* (Rho guanine nucleotide exchange factor 10), *ATP11A* (ATPase phospholipid transporting 11A), *HDAC4* (histone deacetylase 4), *PRR4* (proline rich 4), *PTPRD* (protein tyrosine phosphatase, receptor type D), and *TBCD* (tubulin folding cofactor D)) in MDA-MB-231 cells induced by irradiation exposure are enriched in pathways related to control of the cell cycle, DNA repair, and apoptosis. 

#### 2.3.9. Cancer Stem Cells

As intrinsic cancer stem cells (CSCs) are responsible for radioresistance and metastasis in various cancer types [2,66,67,68], radiation-induced alterations in DNA methylation in this cell population are particularly interesting. A recent report showing that mouse embryonic stem cells do not exhibit changes in DNA methylation levels after radiation suggests that global levels of methylation in stem cells are not determinants of radiosensitivity [69]. However, alterations in DNA methylation levels were observed in offspring of radiation-treated mice [70,71]. These findings indicate that epigenetic changes are transmitted through the germline and cause genomic destabilization, representing a possible cause of cancer [70]. Another study combined irradiation with 5-aza-2′-deoxycytidine to improve the cancer killing effects through inhibiting proliferation and promoting apoptosis of pancreatic cancer cells, both in vitro and in vivo. Interestingly, this combinatorial effect was preferentially targeted to pancreatic CSCs through inhibition of regulatory factors of self-renewal and surface markers. Further experiments revealed significant downregulation of the OCT4 (POU class 5 homeobox 1, POU5F1)-centered transcriptional network of genes in cells in response to the combination treatment. Radiotherapy in combination with DNA methylation inhibitors may, therefore, present a novel therapeutic anti-cancer strategy [72]. 

### 2.4. Therapeutic Potential of DNA Methylation-Targeted Drugs in Radiotherapy

Accumulating evidence suggests that the DNA methylation landscape influences cellular responses to irradiation. This knowledge may be further utilized in modulation of the response of normal and cancerous cells to irradiation, as well as application of DNA methylation-targeted drugs as radiosensitizers.

Based on the finding that depletion of DNMTs results in global demethylation [73], several DNMT inhibitors, including the nucleoside analogs 5-azacytidine (5-aza), 5-aza-2′-deoxycytidine (decitabine), and zebularine, have been successfully used for hematological malignancies, and are currently under trial for treatment of several solid tumors [74,75,76]. Inhibitors of DNMT are nucleoside analogs that irreversibly bind DNMTs to DNA, thereby inhibiting their function [77]. Thus, the genes silenced via methylation can be rescued. Inhibitors of DNMT are hypothesized to influence radiosensitivity through several mechanisms. For instance, as blockers of DNA synthesis, DNMT inhibitors suppress not only DNA repair, but also the number of tumor clonogens, exerting cytotoxicity to proliferative cells following radiotherapy. Additionally, these compounds are considered a trigger of apoptosis [78]. Mounting evidence indicates that utilization of nucleoside analogs increases the sensitivity of various cancer types to radiotherapy, including gastric [79], colorectal [80], head and neck, nasopharyngeal [80], and brain cancer [81]. Moreover, removal of DNMT inhibitors led to recovery of radioresistance to previous levels for all cancer types, except DNMT-deficient tumor cells. For instance, HCT116, a colorectal cell line deficient in DNMT3B and DNMT3B/DNMT1^−/−^, displayed a trend of increased radiosensitivity, which was not observed in DNMT1-deficient cells [80]. 

As aza and decitabine are relatively toxic to normal cells and cannot be administered orally, other DNMT inhibitors, such as zebularine and 5-fluoro-2′-deoxycytidine, have been developed [77] that are also closely related to radiosensitivity [78,81]. 

Despite the promising results, the majority of data have been generated from in vitro studies so far, and several concerns regarding the safety of radiation treatment in combination with DNMT inhibitors to healthy tissue need to be addressed. Demethylating agents also cause hypomethylation of normal tissue, which may influence radiation-induced abscopal effects, risk of secondary tumor development, and virus reactivation (e.g., Epstein-Barr virus) [80,82]. Moreover, a number of in vitro and in vivo studies have indicated that AZA and decitabine cause chromosomal instability, decreased fertility, and loss of offspring [78]. To address these issues, non-nucleoside inhibitors have recently been developed that target DNMT directly without incorporation into DNA. These compounds display demethylating activity both in vitro and in vivo, and are currently undergoing clinical trials [81]. Further in vivo and clinical studies are required to resolve the multiple problems and clarify the biological mechanisms underlying the effects of DNMT inhibitors on radiosensitivity.

## 3. Roles of RNA m^6^A Methylation in Radiotherapy and Cancer Progression

Analogous to DNA methylation, RNA methylation occurs at the *N*^6^ position of adenosine (m^6^A) of transcripts, leading to context-dependent perturbations in the duplex structure. m^6^A, initially identified in 1974 [83], is the most widespread base modification of all RNA types, including mRNA, ncRNA, snoRNA, tRNA, rRNA, and others, accounting for ~0.2–0.6% of all adenosines in mammalian mRNA and over three sites within a G(m^6^A)C (70%) or A(m^6^A)C (30%) consensus sequence per transcript [84,85,86]. m^6^A on mRNA is installed, recognized, and erased post-transcriptionally through m^6^A methyltransferases (writer) [87,88,89,90,91], demethylases (eraser) [92,93], and m^6^A-specific binding proteins (readers) [94,95]. High-throughput sequencing revealed that distribution of m^6^A in mature transcripts is not random, but mainly occurs in the 5′, 3′ untranslated regions (5′-, 3′-UTR) and within internal long exons [96,97,98], consequently influencing the procession and function of RNAs, including RNA stability [99,100], mRNA translation [101,102,103], alternative splicing [104,105] and polyadenylation [106]. Due to the crucial roles of RNA in genetic regulation, RNA m^6^A patterns play important roles in regulating biological functions of mammalian and cancer cells [12,107,108]. Various issues on RNA methylation and demethylation in relation to cancer therapy and progression remain to be clarified. Recently, m^6^A in RNA was shown to be involved in the DNA damage response following irradiation [17]. Since the treatment effects of radiation mainly rely on DNA damage, m^6^A in RNAs involved in the damage response and repair processes of tumor cells potentially have significant effects on the outcomes of radiotherapy. 

CSCs are a crucial contributor to radioresistance and disease recurrence after radiotherapy in the majority of cancers [2,3,4]. Notably, m^6^A modification is involved in CSC generation and radioresistance of tumor cells. Thus, manipulation of inhibitors or inducers of m^6^A modifications could be advantageous in the treatment of radioresistant tumor cells [109]. 

### 3.1. m^6^A Methyltransferases (m^6^A Writers)

m^6^A formation within mRNA is catalyzed by the m^6^A writers containing catalytically active METTL3 (methyltransferase-like 3)–METTL14 (methyltransferase-like 14) complex and other associated proteins [87]. WTAP (Wilms’ tumor 1-associating protein), VIRMA (vir-like m^6^A methyltransferase associated), RBM15 (RNA binding motif protein 15), and RBM15B/OTT3 (RNA-binding motif protein 15B) are established partners of METTL3. METTL3 contains a catalytically active methyltransferase domain that methylates mRNA, but not rRNA [110]. METTL14 is an active component of the m^6^A methyltransferase complex that forms a heterocomplex with METTL3 [91,111]. Biochemical analyses have revealed that METTL3 and METTL14 form a stable complex at a stoichiometric ratio of 1:1 [112]. The methylation activity of METTL14 is only slightly higher than that of METTL3 in vitro. Interestingly, however, the heterodimer of METTL3 and METTL14 exhibits enhanced methylation efficiency. In addition, METTL14 serves as an RNA adaptor protein to enhance the methyltransferase activity of the m^6^A writer complex.

Homologous genes of WTAP were initially identified in *Arabidopsis thaliana* and yeast, and shown to be associated with the METTL3–METTL14 complex [113,114]. This m^6^A writer localizes in nuclear speckles to participate in RNA methylation and processing [88,91,115,116]. Due to the lack of an active catalytic methylation domain, WTAP does not possess methylation activity, but interacts with METTL3–METTL14 heterodimer to influence the RNA m^6^A load in cells. 

RBM15 and its paralog RBM15B associate with METTL3 in a WTAP-dependent manner [92,117]. Both RBM15 and RBM15B contain RNA-binding domains that facilitate writer complex binding to specific mRNAs. For instance, RBM15 and RBM15B guide the m^6^A-methylation complex to lncRNA X-inactive specific transcript (XIST) for consequent repression [118]. 

Recently, METTL16 was identified as an RNA methyltransferase that exerts its functions independently of the m^6^A writer complex containing METTL3 [119,120]. The protein acts as a conserved U6 snRNA methyltransferase, and displays an additional function in vertebrates in regulating the homeostasis of *S*-adenosylmethionine (SAM) by modulating alternative splicing of *MAT2A* (methionine adenosyltransferase 2A) through differential methylation of mRNA hairpin loops [119]. 

### 3.2. m^6^A Demethylase (m^6^A Erasers)

Fat mass and obesity-associated protein (FTO) is a member of the AlkB (Alkylation repair homologs) subfamily of Fe(II)/alpha-ketoglutarate-dependent dioxygenases, and was originally described as an eraser of m^6^A modifications in RNA. FTO was initially reported to demethylate single-stranded DNA (ssDNA) and single-stranded RNA (ssRNA) via removal of *N*^3^-methylthymidine [121,122]. Further experiments revealed that silencing of FTO in HeLa and 293FT cells leads to an increase in total m^6^A in polyadenylated RNA, while its overexpression causes a decrease in m^6^A levels [92,117]. Recently, FTO was identified as an eraser for the *N*^6^, 2′-*O*-dimethyladenosine (m^6^Am) modification co-detected with m^6^A [123]. Advanced detection techniques could effectively differentiate between m^6^A and m^6^Am to facilitate detailed examination of the substrate spectrum of FTO. Biochemical analyses revealed that m^6^Am is the preferred cellular target of FTO in vivo [123]. FTO contains a unique C-terminal domain, distinct from that of other proteins in the AlkB family [121,124], which engages in additional protein–RNA and protein–protein interactions that influence the function of the protein [125,126]. 

ALKBH5 (AlkB homolog 5) is another mammalian demethylase belonging to the AlkB subfamily with efficient demethylation activity on m^6^A in mRNA. ALKBH5 contains an alanine-rich sequence and potential coiled–coil structure within the N-terminal region that is responsible for its nuclear localization [126]. Structural analyses revealed a putative region contributing to binding of dsDNA. Moreover, silencing of ALKBH5 is reported to impair both mRNA export and processing in nuclear speckles [99,101,127,128]. ALKBH5 participates in multiple physiological functions, including fertility, cell survival, and apoptosis, via regulating stability, splicing, subcellular localization, and translation efficiency of mRNA [12,84,93,129]. 

### 3.3. m^6^A Binding Proteins (m^6^A Readers) 

m^6^A methylations of RNAs are mainly read by eukaryotic initiation factor 3 (eIF3) and proteins containing a YT521-B homology (YTH) domain belonging to the YTH protein family. However, several RNA binding proteins (RBP) that associate with m^6^A do not belong to the classical m^6^A YTH domain family. YTHDF1 (YTH *N*^6^-methyladenosine RNA binding protein 1), YTHDF2 (YTH *N*^6^-methyladenosine RNA binding protein 2), YTHDF3 (YTH *N*^6^-methyladenosine RNA binding protein 3), YTHDC1 (YTH domain containing 1), and YTHDC2 (YTH domain containing 2), members of the YTH domain family, represent the predominant cytoplasmic m^6^A reader proteins [94]. Proteins of the YTH domain family preferentially bind RNA with m^6^A at the (G>A)m^6^ACU consensus sequence, compared to unmethylated RNA of the same transcript [95]. YTHDF2 is the first characterized m^6^A reader protein shown to mediate RNA decay by targeting RNA substrates to P-bodies in an m^6^A-dependent manner [130,131]. YTHDF1 and YTHDF3 are proposed to modulate translation machinery, and consequently influence the translation efficacy of target mRNAs [132,133,134]. 

Nuclear YTH domain-containing 1 (YTHDC1), also designated YT521-B, has been identified as a nuclear m^6^A reader that binds m^6^A through tryptophan residues at positions 377 and 428 by forming an aromatic cage [135]. A glutamic acid-rich region of the amino terminus and glutamic acid/arginine-rich regions of the carboxyl terminus of YTHDC1 are responsible for nuclear localization and formation of YT bodies [136]. Due to the proximity of YT bodies to nuclear speckles, YTHDC1 may access nascent mRNA, and subsequently facilitate recruitment of RNA splicing factors to regulate pre-mRNA splicing [137]. 

Nuclear YTH domain-containing 2 (YTHDC2) was initially reported as a factor required for HCV genome replication [138]. This factor is widely expressed in human cells and shown to promote cancer metastasis through enhancing translation of hypoxia-inducible factor-1α (HIF-1α) and TWIST (Twist family bHLH transcription factor 1) [139]. Recently, YTHDC2 was reported as an m^6^A reader essential for male and female fertility in mice via regulation of the m^6^A transcriptome. Additionally, this factor is highly expressed in germ cells, and interacts with an essential meiosis-specific protein, MEIOC (meiosis specific with coiled-coil domain) [140].

Other than the YTH domain family, pulldown assays revealed that heterogeneous nuclear ribonucleoprotein A2/B1 (HNRNPA2B1) binds m^6^A in the nucleus [94]. Another study consistently demonstrated that HNRNPA2B1 interacts with the m^6^A site of RNA transcripts, and regulates splicing and maturation of microRNA (miRNA) [141]. 

Eukaryotic initiation factor 3 (eIF3), a protein complex that functions in the initiation of eukaryotic translation, has additionally been identified as an important m^6^A binding protein. Following binding to m^6^A in the 5′-UTR of RNA transcripts, initiation of translation can be triggered by eIF3 in a 5’cap- and eukaryotic initiation factor 4E (eIF4E)-independent mechanism [142]. These findings support an alternative mechanism of translational initiation mediated via m^6^A modifications in 5′-UTRs of mRNA when eIF4-dependent initiation is hindered by specific cell states. Furthermore, m^6^A has been shown to influence the secondary structure of RNAs through binding of regulatory proteins, consequently modulating the expression or maturation of RNA transcripts [143,144,145]. 

For instance, secondary structure changes induced via m^6^A modification facilitate binding of HNRNPC (heterogeneous nuclear ribonucleoprotein C) and RBMX (RNA binding motif protein, X-linked) for targeting and regulation of mRNA expression and splicing [146]. 

### 3.4. m^6^A-Mediated Cancer Progression or Radioresistance 

#### 3.4.1. Glioblastoma Multiforme (GBM) 

Glioblastoma multiforme (GBM) is the most aggressive glioma type, affecting ~17,000 patients per year. Treatment failure of glioblastoma may be due to tumor heterogeneity and treatment resistance of cancer stem cells, triggering disease recurrence [22,147]. Several studies have indicated that m^6^A in RNA is associated with tumorigenesis and progression of glioblastoma [109,148]. Overexpression of METTL3 or inhibition of the RNA demethylase, FTO, is known to suppress GSC growth and self-renewal. Moreover, suppression of FTO causes tumor regression and promotes survival rates in GSC-grafted mice. Several oncogenes, including *ADAM19* (ADAM metallopeptidase domain 19), *EPHA3* (EPH receptor A3), and *KLF4* (Kruppel like factor 4), are upregulated in METTL3 or METTL14-depleted GSCs, and conversely downregulated in GSCs with METTL3 or METTL14 overexpression [109]. 

METTL3-mediated m^6^A modification is proposed to play a crucial role in glioma stem-like cell (GSC) maintenance and dedifferentiation of glioma cells [16], based on the finding that expression of METTL3 is elevated in GSCs and attenuated in differentiated cells. Further experiments demonstrated that *SOX2* is the m^6^A target of METTL3. Recruitment of human antigen R (HuR) to m^6^A sites was essential for *SOX2* mRNA stabilization by METTL3. Notably, silencing of METTL3 enhanced sensitization of GSC to radiotherapy, and expression levels of METTL3 predicted poor survival in GBMs enriched for GSC-specific signatures [16]. Zhang et al. [148] recently reported that elevated expression of ALKBH5 in GSCs is predictive of poor patient prognosis. Moreover, ALKBH5 demethylated nascent *FOXM1* (forkhead box M1) transcripts, consequently enhancing *FOXM1* expression. *FOXM1-AS*, a lncRNA that localizes in the nucleus, has been shown to promote interactions between ALKBH5 and nascent *FOXM1* transcripts. Silencing of ALKBH5 and *FOXM1-AS* inhibited the tumorigenic potential of GSCs via suppression of FOXM1. Thus, m^6^A modifications could influence the expression or function of genes associated with the malignant phenotype of cancer, presenting a promising therapeutic target in the tumor. 

#### 3.4.2. Acute Myeloid Leukemia (AML)

Leukemias are a clinically and genetically heterogeneous group of hematopoietic disorders that begin in early blood-forming cells found in the bone marrow. Acute myeloblastic leukemia (AML) is more frequent in older patients, with diagnosis at about 70 years of age on average [149]. Genetic and epigenetic regulation processes play crucial roles in classification, risk stratification, and management of acute leukemia types [150]. Increasing numbers of gene mutations, dysregulation, and epigenetic alterations have been associated with progression of acute leukemia [151,152]. FTO, an m^6^A eraser, promotes leukemic oncogene-mediated cell transformation and leukemogenesis, and suppresses all-trans-retinoic acid (ATRA)-triggered AML cell differentiation by regulating the expression of specific target genes, including ankyrin repeat and SOCS box-containing 2 (*ASB2*) and retinoic acid receptor a (*RARA*), through repressing m^6^A levels in these transcripts [153]. *ASB2* (ankyrin repeat and SOCS box containing 2) and *RARA* (retinoic acid receptor alpha) are induced during hematopoiesis and function as crucial regulators of ATRA-induced differentiation of leukemia cells. Furthermore, inhibition of FTO-mediated ASB2 and RARA suppression contributes to the response of AML cells to ATRA treatment [153]. Another report suggests that isocitrate dehydrogenase 1 or 2 (IDH1/2) mutant tumors account for ~20% of AML cases, causing aberrant metabolite D-2-hydroxyglutarate (D2HG) production [154], which acts as a competitive inhibitor of FTO through suppressing the activities of alpha-ketoglutarate-dependent enzymes. In a further in vitro study, cells expressing the IDH2 mutant contained significantly higher levels of m^6^A in RNA transcripts than isogenic IDH2 wild type expressing cells. Therefore, the precise roles of FTO in the pathogenesis of AML require elucidation in the context of the IDH1/2 mutation in this setting [155]. 

Recently, a role for METTL3 in regulating myeloid differentiation and maintaining myeloid leukemia has been reported [156,157]. Both mRNA and protein levels of METTL3 are more highly expressed in AML cells than hematopoietic stem and progenitor cells (HSPC). Additionally, depletion of METTL3 in human myeloid leukemia cell lines induces cell differentiation and apoptosis, while suppressing leukemia progression in recipient mice in vivo. Analysis of single nucleotide resolution mapping of m^6^A, in combination with ribosome profiling, revealed that METTL3-mediated m^6^A modification promotes translation of *c*-*MYC* (MYC proto-oncogene, bHLH transcription factor), *BCL-2* (B cell leukemia/lymphoma 2), and *PTEN* (phosphatase and tensin homolog) in human AML cells [156]. Another study confirmed the important roles of METTL3 in leukemia progression. The authors showed that METTL3 associates with chromatin, and localizes to the transcriptional start sites of active genes, inducing m^6^A modification within the coding region of the associated mRNA transcripts, and consequently, enhancing its translation [157]. Further analyses disclosed that mutations and copy number variations of m^6^A regulatory genes are strongly associated with the presence of TP53 mutations in AML patients [158]. These results suggest that genetic alterations of m^6^A regulatory genes in cooperation with TP53 contribute to the pathogenesis and maintenance of AML, and provide a rationale for therapeutic targeting of METTL3 in myeloid leukemia. 

#### 3.4.3. Lung Cancer 

Lung cancer is the most commonly diagnosed cancer type and the leading cause of cancer-related mortality worldwide, with a <15% 5-year survival rate, despite the progressive improvements in standard therapies [159]. METTL3 is significantly upregulated in lung cancer, in turn, promoting cellular proliferation, survival, and metastasis. In human lung cancer cells, METTL3 promotes the translation of specific genes, such as *EGFR* (epidermal growth factor receptor) and Hippo pathway effector, *TAZ* (tafazzin), through recruitment of eIF3 to the translation initiation complex. Although m^6^A is implicated in regulation of translation through binding YTHDF1 [134], another model has been proposed in which METTL3 enhances both cap-binding proteins 80 and 20 (CBP80/20), and eIF4E-dependent translation via binding to a specific subset of transcripts, and helping to recruit eIF3 to translation. Moreover, an independent pathway of mRNA–ribonucleoprotein complexes’ remodeling may entail replacement of METTL3 with YTHDF1 at the m^6^A methylated sites, and enhance the translation of a distinct subset of genes, including those encoding EGFR and TAZ. 

#### 3.4.4. Hepatocellular Carcinoma (HCC) 

HCC is the fifth most prevalent malignant tumor, and third leading cause of cancer-related deaths worldwide [160]. Recent investigations of m^6^A-related mechanisms in HCC have provided new ideas for treatment. Silencing of METTL14 decreases the m^6^A level in RNA transcripts and enhances cancer metastasis, both in vitro and in vivo [161], and downregulation of METTL14 in HCC serves as an adverse prognostic factor for recurrence-free survival. METTL14 has been shown to interact with the microprocessor protein, DGCR8 (DGCR8, microprocessor complex subunit), and modulate primary processing of microRNA-126 in an m^6^A-dependent manner. These findings suggest that METTL14 participates in the regulation of microRNAs in tumor biology, and highlight its potential as a therapeutic target in HCC [161]. 

In addition, METTL3 is overexpressed in HCC, and associated with poor prognosis. Silencing of METTL3 is reported to suppress tumorigenesis and progression of HCC, and conversely, its overexpression significantly promotes HCC growth, both in vitro and in vivo. Further experiments revealed that METTL3 induces m^6^A modification of the *SOCS2* transcript, and consequently decreases its stability in a YTHDF2-dependent manner [162]. 

Another recent study demonstrated that YTHDF2 expression is closely associated with malignancy of HCC, and negatively correlated with miR-145 [163]. Bioinformatics and functional assays further revealed that miR-145 targets the 3′-UTR of *YTHDF2* mRNA, and inhibits its expression in HCC. Moreover, overexpression of miR-145 induced a strong increase in m^6^A levels which could be blocked upon YTHDF2 overexpression [163]. These studies strongly suggest that not only methyltransferases and demethyltransferases, but also readers of m^6^A, control the global m^6^A level in cells to influence cancer progression. 

#### 3.4.5. Breast Cancer

Breast cancer is the most common cancer type, and the fourth leading cause of cancer-related deaths among women worldwide [164]. Tumor hypoxia, a common phenomenon in the majority of malignant tumors, is a condition whereby tumor cells are deprived of oxygen, causing advanced but dysfunctional vascularization, and acquisition of epithelial-to-mesenchymal transition phenotype that results in cell metastasis, the major cause of breast cancer related mortality [165]. Exposure of breast cancer cells to hypoxia promotes the ALKBH5-mediated demethylation of m^6^A in *NANOG* transcripts, consequently enhancing *NANOG* expression. NANOG is a potent pluripotency factor that stimulates CSC maintenance. Similar to hypoxia exposure, overexpression of ALKBH5 not only decreases *NANOG* mRNA methylation, but also increases *NANOG* (Nanog homeobox) levels, resulting in elevation of the CSC population in breast cancer [166]. Exposure to hypoxia also induces ZNF217 (zinc finger protein 217)-dependent inhibition of m^6^A methylation of *NANOG* and *KLF4* transcripts, both important pluripotency factors that mediate CSC maintenance, in turn, influencing cancer radiosensitivity and recurrence [167]. Therefore, hypoxia of cancers may stimulate pluripotency factor expression and CSC maintenance through regulation of RNA methylation. 

#### 3.4.6. Renal Cell Carcinoma (RCC) 

Renal cell carcinoma (RCC) is currently the ninth most common cancer type in men and 14th most common in women globally [168]. The mortality rate of RCC has continuously increased by ~1.5–5.9% per year [169]. Recent experiments showed that METTL3 is downregulated in clinical specimens of RCC, and negatively associated with larger tumor size and higher pathological grade. Notably, RCC patients positive for METTL3 expression had a better survival rate. Moreover, METTL3 was associated with regulation of cellular proliferation, migration, and metastasis through the EMT and PI3K-Akt-mTOR pathways in RCC. The collective findings suggest that loss of METTL3 expression serves as a marker for tumorigenesis, development, and survival of RCC [170]. 

#### 3.4.7. Cervical Cancer

Cervical cancer is one of the most prevalent gynecological malignancies worldwide [171,172]. A recent analysis of m^6^A mRNA methylation levels in 286 pairs of cervical cancer samples disclosed significant downregulation in cervical cancer and negative correlation with characteristics of malignancy, including pathologic stage, tumor size, differentiation, lymph invasion, and cancer recurrence. Suppression of the m^6^A level, via FTO or ALKBH5 overexpression, promoted proliferation of cervical cancer cells, whereas increasing the m^6^A level led to significant suppression of tumor development, both in vitro and in vivo. Thus, inhibition of the m^6^A level is tightly associated with cervical cancer progression, and regulators of m^6^A mRNA methylation present potential therapeutic targets in cervical cancer [173]. 

### 3.5. Clinical Application of m^6^A 

Cancer stem cells (CSC) represent a subpopulation of cancer cells with the capacities of radioresistance, metastasis, and tumor initiation in various cancer types [2,66,67,68]. As crucial regulators of m^6^A in RNAs play important roles in cancer stem cell maintenance and progression, targeting of m^6^A editing may present an effective treatment strategy. For instance, inhibition of the RNA demethylase, FTO, suppresses GSC growth and self-renewal, consequently suppressing tumor progression of GBM. Additionally, FTO exerts a critical oncogenic effect on ATRA-induced leukemic cell differentiation by decreasing m^6^A levels in critical mRNA transcripts, including *ASB2* and *RARA* [153]. Rhein, a natural compound that is neither a structural analog of α-ketoglutaric acid nor a of metal ion-chelator, competitively targets to the active site of FTO. Data from an earlier in vitro study indicate that Rhein exerts inhibitory activity on m^6^A demethylation within cells [100]. Meclofenamic acid (MA) has been developed as another selective inhibitor of FTO that acts via competitive binding to m^6^A-containing regions in RNAs [102]. Moreover, MA2, an ethyl ester form of MA approved by US Food and Drug Administration (FDA) as a nonsteroidal anti-inflammatory drug, serves as a competitive inhibitor of FTO [102]. Thus, Rhein, MA, or MA2 may induce tumor regression in GBM, or increase ATRA efficacy in AML through inhibiting the RNA demethylase FTO. Furthermore, citrate and IOX3 serve as inhibitors of ALKBH5, another m^6^A demethylase that maintains tumorigenicity of glioblastoma stem cells [99,127]. However, the specificities of these inhibitors need to be validated in vivo, and development of selective inhibitors of other m^6^A regulators should aid in overcoming disease recurrence and resistance to cancer therapy. In addition to FTO and ALKBH5, *S*-adenosylhomocysteine (SAH) acts as a kind of competitive inhibitor of adenosylmethionine-dependent methyltransferases [103]. SAH is hydrolyzed by SAH hydrolase into adenosine and homocysteine, which are important regulators of intracellular SAH levels. 3-Deazaadenosine (DAA), one of the most frequently used SAH hydrolysis inhibitors, has been shown to suppress m^6^A methylation of mRNA substrates [174]. Moreover, DAA and its analogs can suppress the replication of various viruses that are capable of extensive editing of m^6^A-containing mRNA [106,175]. The effects of DAA on cancer therapy are yet to be investigated in detail. 

## 4. Conclusions

Radiotherapy is one of the major forms of cancer treatment for various malignant tumor types. Radiation treatment triggers DNA damage through ionization or production of reactive oxygen species (ROS), leading to death of tumor cells, but can concomitantly promote radioresistant cancer cell metastasis and progression [22]. Accumulating evidence suggests that epigenetic alterations play important roles in radioresistance and cancer recurrence. Epigenetic approaches, including evaluation and manipulation of DNA and RNA methylation patterns, could thus present a crucial strategy to elucidate the biological effects of irradiation on tumors. However, the majority of documented results on the effects of irradiation on methylation of DNA or RNA are derived from in vitro and in vivo experimental systems, and limited investigations have focused on non-cancerous and tumor regions in humans to date. Thus, translational studies need to be conducted to ascertain the clinical impacts of DNA or RNA methylation on radiotherapy. However, radiation treatment is known to cause epigenetic alterations. Irradiation-induced DNA/RNA methylation markers or a marker panel may therefore be effectively utilized to predict outcomes for patients treated with radiotherapy. While no epigenetic drugs have been approved for application in humans as yet, their therapeutic benefits are clearly promising, and could be a prospective radiosensitizer in the clinic. 

## Figures and Tables

**Table 1:** Summary of the relevant DNA methylation of genes associated with radiotherapy/response in various cancers.

Cancer Types	Gene Names	Methylation Status	Response to RT	Irradiation Induced Methylation Alteration	Prognostic Marker of Cancer	Reference
Glioblastoma multiforme	*MGMT*	Hyper	Sensitive		●	[43,44,45]
Lung cancer	*CDKN2A*	Hyper	Resistant	Hyper		[46,47]
	*SERPINB5* & *S100A6*	Hyper	Resistant			[48]
	*CAT* & *BNCI*	Hypo				
Esophageal cancer	*CDKN2A*, *RPRM*, *CDKN1C*, *TP73*, *RUNX3*, *CHFR*, *MGMT*, *TIMP3* & *HPP1*	Hypo	Sensitive		●	
					●	[49,50]
Cervical cancer	*TP73*	Hyper	Resistant			[51]
Head and Neck squamous cell carcinoma	*TIMP3* & *CDH1*	Hyper	Sensitive		●	[52]
	*IRX1*, *EBF3*, *SLC5A8* & *FUSSEL18*			Hyper		[40]
	*CCND2*	Hyper	Resistant			[53]
Nasopharyngeal carcinoma	miR-24 & *FHIT*	Hyper	Resistant			[54]
			Resistant		●	[55]
Laryngeal cancer	*TOPO2A*, *PLXDC2*, *ETNK2*, *GFI1* & *IL12B*	Hyper	Resistant			[43]
Breast cancer	*ADAMTS9*, *FOXC1* & *TRAPPC9*			Hyper		[39]
	*AMIGO3*			Hypo		
	*AGAP1*, *ARHGEF10*, *ATP11A*, *HDAC4*, *PTPRD*, *PRR4* & *TBCD*			Hyper		[33]

●: determined; RT: radiotherapy.
